# Sodium Influx and Potassium Efflux Currents in Sunflower Root Cells Under High Salinity

**DOI:** 10.3389/fpls.2020.613936

**Published:** 2021-01-18

**Authors:** Palina Hryvusevich, Ilya Navaselsky, Yuliya Talkachova, Darya Straltsova, Monika Keisham, Aliaksei Viatoshkin, Veranika Samokhina, Igor Smolich, Anatoliy Sokolik, Xin Huang, Min Yu, Satish Chander Bhatla, Vadim Demidchik

**Affiliations:** ^1^International Research Centre for Environmental Membrane Biology, Foshan University, Foshan, China; ^2^Department of Plant Cell Biology and Bioengineering, Biological Faculty, Belarusian State University, Minsk, Belarus; ^3^Laboratory of Plant Physiology and Biochemistry, Department of Botany, University of Delhi, New Delhi, India

**Keywords:** sunflower, nonselective cation channels, potassium channels, salinity, calcium, salt stress

## Abstract

*Helianthus annuus* L. is an important oilseed crop, which exhibits moderate salt tolerance and can be cultivated in areas affected by salinity. Using patch-clamp electrophysiology, we have characterized Na^+^ influx and K^+^ efflux conductances in protoplasts of salt-tolerant *H. annuus* L. hybrid KBSH-53 under high salinity. This work demonstrates that the plasma membrane of sunflower root cells has a classic set of ionic conductances dominated by K^+^ outwardly rectifying channels (KORs) and non-selective cation channels (NSCCs). KORs in sunflower show extreme Na^+^ sensitivity at high extracellular [Ca^2+^] that can potentially have a positive adaptive effect under salt stress (decreasing K^+^ loss). Na^+^ influx currents in sunflower roots demonstrate voltage-independent activation, lack time-dependent component, and are sensitive to Gd^3+^. Sunflower Na^+^-permeable NSCCs mediate a much weaker Na^+^ influx currents on the background of physiological levels of Ca^2+^ as compared to other species. This suggests that sunflower NSCCs have greater Ca^2+^ sensitivity. The responses of Na^+^ influx to Ca^2+^ correlates well with protection of sunflower growth by external Ca^2+^ in seedlings treated with NaCl. It can be, thus, hypothesized that NaCl tolerance in sunflower seedling roots is programmed at the ion channel level *via* their sensitivity to Ca^2+^ and Na^+^.

## Introduction

Sunflower (*Helianthus annuus* L.) is an important crop that is widely used in the oil industry and animal feeding. Global sunflower production increased more than twice since 2000 (Pilorgé, 2020). It is the third highest oilseed produced in the world, the fourth vegetable oil and the third protein feed source among oilseed crops. Although sunflower plants exhibit medium salt tolerance, their production is affected by high soil salinity, which is common in arid or semi-arid areas (Karrenberg et al., 2006; Li et al., 2020). Understanding response to salinity in sunflowers and, in particular, the molecular basis of its salt tolerance mechanism is central to development of breeding and bioengineering strategies aimed to improve the salt tolerance in this species.

The major toxic ions in salinized soils are Na^+^ and Cl^−^, although SO_4_^2−^, HCO_3_^−^, Mg^2+^, and other chemical species also contribute to the harmful salinity effects among higher plants. Slower growth is observed in plant species in the presence of external 40–50 mM NaCl, while the treatment by 100–150 mM NaCl is usually lethal (Flowers et al., 1986; Munns and Tester, 2008). Some species and cultivars tolerate up to 200–400 mM NaCl, and they are considered salt-tolerant or halophytic species (Shabala, 2013; Flowers and Colmer, 2015). Agriculturally important *H. annuus* L. cultivars survive at 100–150 mM NaCl while some wild sunflower species, such as halophytic *Helianthus paradoxus*, can withstand much higher levels of NaCl (Karrenberg et al., 2006; Grieve et al., 2012).

The physiological response to salinity is a complex phenomenon that normally includes rapid Na^+^ influx in root cells, triggering early signaling and defense reactions and longer-term NaCl-induced processes which develop within 1–2 weeks, and subsequently target photosynthetic tissues as the plants grow (Munns and Tester, 2008; Flowers et al., 2014; Negrão and Schmöckel, 2017). High levels of Na^+^ and Cl^−^ do not have specific cellular targets. Therefore, NaCl toxicity is related to a long-term and non-specific disturbance of cellular osmotic, ionic, electric, redox and metabolic balance, inhibiting photosynthesis, respiration, growth, development and reproduction (Shabala, 2013; Demidchik et al., 2014, 2018; Negrão and Schmöckel, 2017; Isayenkov and Maathuis, 2019). It is generally accepted that Na^+^ is more toxic than Cl^−^ for most plant species (Shabala, 2013; Flowers and Colmer, 2015), although the detrimental effect of Cl^−^ should not be underestimated (White and Broadley, 2003; Li et al., 2020). The key mechanisms of toxic Na^+^ influx into the root cells is its entry through the plasma membrane-associated non-selective cation channels (NSCCs; Demidchik and Tester, 2002; Shabala et al., 2006; Demidchik and Maathuis, 2007). However, the existence of other pathways, such as HKT1-type transporters and K^+^-selective channels, has also been hypothesized (Xue et al., 2011; Flowers et al., 2014; Assaha et al., 2017). Influx of Na^+^ through NSCCs results in plasma membrane depolarization, which is probably the earliest physiological response to NaCl, leading to the activation of Ca^2+^ influx, K^+^, and anion efflux and water release for osmotic balance (Demidchik and Tester, 2002; Shabala et al., 2006). Na^+^ influx-induced depolarization triggers the generation of reactive oxygen species (ROS) catalyzed by nicotinamide adenine dinucleotide phosphate (NADPH) oxidases and other redox systems which additionally stimulate Ca^2+^ influx and loss of K^+^ (as well as the loss of other electrolytes), leading to a long-term ionic and redox disequilibrium, which is considered to be the prime reason for NaCl toxicity among most plants (Demidchik et al., 2014; Demidchik and Shabala, 2018).

Na^+^ influx through NSCCs can be inhibited by increased external [Ca^2+^] (Demidchik and Tester, 2002). This phenomenon is widely used in agriculture to ameliorate NaCl toxicity (Bressan et al., 1998). We have previously found that high external Ca^2+^ levels inhibit both Na^+^ entry and K^+^ efflux channels, thereby blocking both Na^+^ toxic influx and loss of K^+^ (Shabala et al., 2006). In the recent past, blockade of Na^+^ influx by external Ca^2+^ has only been investigated in *Arabidopsis thaliana* (Demidchik and Tester, 2002; Shabala et al., 2006). Therefore, it is still unclear whether other plants share this mechanism.

In the present investigations, using patch-clamp electrophysiology, we have characterized the NSCC-like Na^+^ conductance and determined its Ca^2+^ sensitivity in root protoplasts of *H. annuus* L. seedlings (hybrid KBSH-53), which is widely cultivated in arid regions of India. To our knowledge, this is the first electrophysiological study of any ion currents in sunflower as well as properties of Na^+^ influx and K^+^ efflux conductances in this species.

## Materials and Methods

### Plant Material

*Helianthus annuus* L. (cv Karnataka Bangalore Sunflower HyBrid 53, KBSH-53) was from the collection of Department of Botany, University of Delhi (India). For patch-clamp experiments, sunflower seeds were surface-sterilized with 20% (w/v) Domestos bleach, germinated on wetted filter paper (2 days), and then cultivated vertically in filter paper rolls immersed in solution containing 10% standard Murashige and Skoog nutrient medium (MS; Duchefa #M0221, original composition; Murashige and Skoog, 1962), pH 6.0 was adjusted by KOH. Six-to-twelve day-old seedlings were used for patch-clamp experiments. Root protoplasts were initially isolated using techniques developed by Demidchik and Tester (2002). Three-cm-long root tips from 10 to 15 seedlings were excised, chopped into small pieces (1–2 mm) and placed in the solution with cellulytic enzymes for protoplasts isolation comprising 1.5% (w/v) Cellulase Onozuka RS (Yakult Honsha, Tokyo, Japan), 1% (w/v) cellulysin (CalBiochem, Nottingham, United Kingdom), 0.1% (w/v) pectolyase Y-23 (Yakult Honsha, Tokyo, Japan), 0.1% (w/v) bovine serum albumin (Sigma), 10 mM KCl, 10 mM CaCl_2_, 2 mM MgCl_2_, 2 mM MES, pH 6.0 with Tris, and 600 mOsm kg^−1^ adjusted with D-sorbitol. This protocol was adjusted as indicated in “Results” section. After gentle shaking (45 rpm) in the enzyme solution for 30–50 min at 28°C, protoplasts were filtered (30-μm pore mesh) and rinsed with holding solution (HS: 5 mM KCl, 2 mM CaCl_2_, 1 mM MgCl_2_, 10 mM sucrose, 10 mM glucose, 2 mM MES, pH 6.0 with Tris, and 600 mOsm kg^−1^ with D-sorbitol). Protoplasts were collected by 5-min centrifugation at 200 *g* and stored on ice in holding solution.

The hydroponic cultivation system was used for sunflower root growth measurements. Germinated seeds (germination: 2 days on wetted filter paper) were cultivated during 7 days in vertical polycarbonate sheets. Each root was directed to a separate channel of polycarbonate sheets in order to prevent root entanglement (Green House Polycarbonate Sheets; Greenhouse Megastore, United States). Polycarbonate sheets were mounted vertically in large square glass vessel and dipped into the medium (volume: 2 L), which was stirred with a stream of air (air compressor Barbus Aquael OXYPRO; China). The medium contained 5% original Murashige and Skoog nutrient composition (MS; Duchefa #M0221; Murashige and Skoog, 1962), рН 6.0 (adjusted by KOH). Treatments (CaCl_2_, NaCl, etc.) were added to this medium as required. All growth solutions were replaced every day (for freshness). Root length (main root) was measured after 7 days of treatment.

### Patch-Clamp Measurements

Conventional patch-clamp and protoplast isolation techniques were used (Demidchik and Tester, 2002; Demidchik et al., 2010). The standard bathing solution contained (in mM): 0.3 KCl, 2 Tris, adjusted to pH 6.0 with 1 MES, and 600 mOsm kg^−1^, with D-sorbitol. Other salines are indicated in figure legends. A freshly prepared mixture of this solution was applied in whole-cell outside-out patches. The pipette solution (PS) contained the following composition (mM): 70 KGluconate, 10 KCl, 1 mM 1,2-bis(o-aminophenoxy)ethane-N,N,N0,N0-tetraacetic acid (BAPTA) and 0.475 mM CaCl_2_ (10 nM free Ca^2+^), pH 7.2 with 2 Tris, and 1 MES. To examine the sensitivity of whole-cell outward current to cation channel blockers (TEA^+^ and Gd^3+^), 10 mM TEACl or 100 μM GdCl_3_ were added to the bathing solution. The size of protoplasts was measured using Nikon NIS-Elements software and used to calculate the mA/m^2^ current densities. Typical transmembrane currents are from the same cell (including Gd^3+^ blockade test). Liquid junction potentials were calculated by JPCalc, which is included in Axon Clampex 10.6 software (Molecular Devices, United States) and corrected. The voltage was held at −90 mV, then square 7.6 s-long or 1.5 s-long depolarizing or hyperpolarizing voltage pulses were applied. Currents were measured using PC-ONE Patch/Whole Cell Clamp (CORNERSTONE Series) amplifier (Dagan Corporation, United States) controlled by Digidata 1,320/Clampex 10.6 (Molecular Devices, United States). Current-voltage (I-V) and other curves were plotted and analyzed using SigmaPlot 10.0 (Systat Software Inc., United States).

## Results

### Effect of Ca^2+^ on Sunflower Seedling Growth in High Salinity Conditions

**Figure 1:**
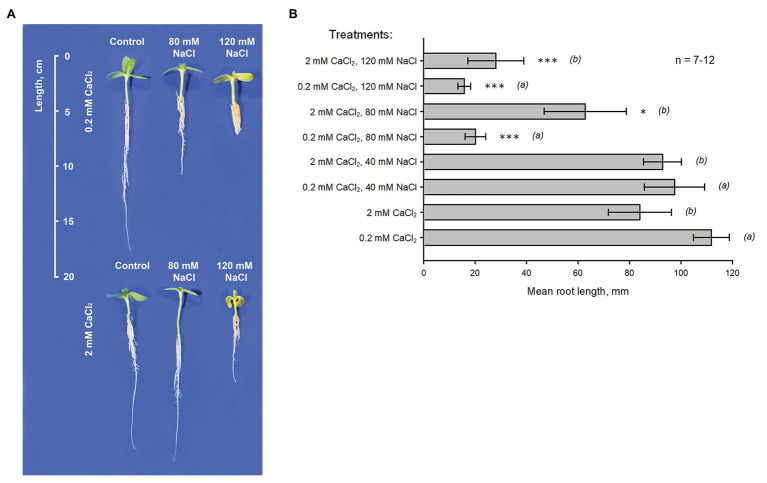
Effect of external Ca^2+^ on the inhibition of *H. annuus* L. root growth induced by NaCl. **(A)** Typical sunflower seedlings cultivated in hydroponics during 7 days (after 2-day germination) on the background of 0.2 or 2 mM CaCl_2_ (upper and lower panels, respectively). “Control”: plants grown in control conditions (5% MS; pH 6.0). “80 mM NaCl” and “120 mM NaCl”: plants grown in the presence of 80 or 120 mM NaCl, respectively. **(B)** Mean ± SE (*n* = 7–12) root length values measured in different conditions (as indicated in the figure). (*a*) and (*b*) are groups for comparison (ANOVA test); ^*^p < 0.01; ^***^p < 0.0001.

Root growth tests were carried out using seedlings of *H. annuus* L. KBSH-53 in vertical hydroponic chambers in controlled environment (Figure 1). The effect of 80 and 120 mM NaCl on the length of main root was examined (preliminary tests showed that 40 mM NaCl did not modify plant growth). Measurements were carried out against two levels of Ca^2+^ (0.2 and 2 mM) in the cultivation solution containing 5% MS (original composition). Growth in NaCl-free solutions (control conditions) containing 2 mM CaCl_2_ was approximately 25% slower than growth on the background of 0.2 mM CaCl_2_ (*p* = 0.007; seven independent trials; each trial included 9–10 plants). Addition of 80 mM NaCl along with 0.2 mM CaCl_2_ resulted in approximately 5-fold decrease of root length (*p* < 0.001; 12 independent trials; each trial included 9–10 plants). At the same time, 120 mM NaCl induced 6.5-fold delay in growth. Increase of external Ca^2+^ level from 0.2 to 2 mM significantly improved plant growth in the presence of NaCl. In this case, application of 80 NaCl did not induce statistically significant decrease of root length (*p* = 0.235; 11 independent trials; each trial included 8–10 plants) while the effect of 120 mM NaCl was twice smaller (*p* = 0.008; eight independent trials; each trial contained eight plants; comparison with 0.2 mM CaCl_2_). Overall, these data show that Ca^2+^ (the physiological range) has a strong ameliorative effect on the growth of *H. annuus* L. KBSH-53 roots in salinized conditions.

### Protoplast Isolation and Obtaining Gigaohmic Resistance Patches

No reports have been published about transmembrane currents of *H. annuus* L. or protocols for protoplast isolation for patch-clamp tests in this species. To our knowledge, several attempts have been made to isolate sunflower protoplasts suitable for patch-clamp studies but none of them were successful for implementation in routine electrophysiological practice. In most cases, protoplast isolation from sunflower required overnight treatment by enzymes and did not yield viable protoplasts from any tissues apart from hypocotyl (Lenee and Chupeau, 1986; Kativat et al., 2012). We have developed protocols for *H. annuus* L. root patch-clamp analyses that were based on previous protocols elaborated for *A. thaliana* and *Triticum aestivum* (Demidchik and Tester, 2002; Demidchik et al., 2010; Straltsova et al., 2015; Sosan et al., 2016; Makavitskaya et al., 2018). Ten osmolality levels were examined (300-750 mOsm kg^−1^; 50 mOsm kg^−1^ step) in 10 replicates. Round shaped viable protoplasts were observed only at 600 and 650 mOsm kg^−1^ but the density of viable protoplasts was approximately six times higher at 600 mOsm kg^−1^ comparing to 650 mOsm kg^−1^ (up to 55 ± 4 viable protoplasts per 1 ml of the enzyme solution; mean ± SE; *n* = 10). Experimental work on protoplasts reported here was carried out using the osmolality level of 600 mOsm kg^−1^.

We have previously developed techniques and voltage-clamp protocols for the patch-clamp analysis of inwardly- and outwardly-directed conductances in higher plants, including Na^+^-conducting NSCCs (Demidchik and Tester, 2002; Demidchik et al., 2010). The probability rate of observing “gigaohmic” contact required for patch-clamp measurements in sunflower protoplasts was low (2,750 protoplasts were patch-clamped; “gigaohmic” contact formed in 409 protoplasts). Approximately one-third of these protoplasts survived after addition of high NaCl concentration and maintained gigaohmic pipette resistance (139 protoplasts). A number of methods for improving patch stability were applied (different levels of external Ca^2+^, H^+^, use of Na^+^ instead K^+^ in the patch-clamp pipette, additional pipette polishing, hydrophobic coating, etc.) but none of these significantly improved the “gigaseal.”

### Currents of Sunflower Root Protoplasts in Control Conditions and in Presence of NaCl

**Figure 2:**
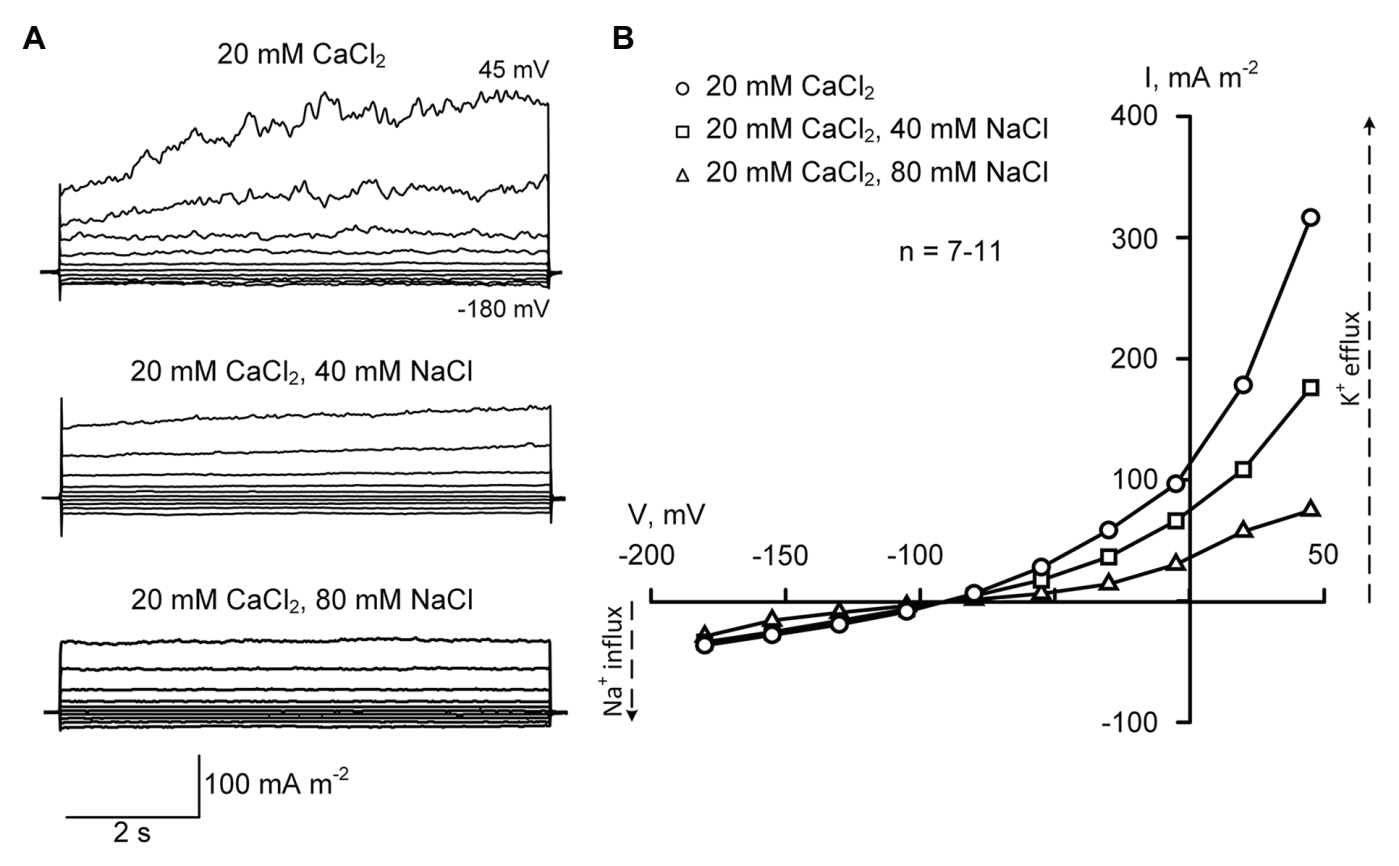
Typical plasma membrane currents **(A)** and mean current-voltage curves **(B)** in *Helianthus annuus* L. root protoplasts recorded in the presence of 20 mM CaCl_2_ in the bathing solution at different levels of external NaCl (40 and 80 mM). Current-voltage curves were plotted by mean values (*n* = 7–11; errors are not shown in the figure). Statistically significant (*p* < 0.01) difference between mean currents was found at voltage values exceeding −30 mV (ANOVA test). The standard bathing solution contained (in mM): 0.3 KCl, 2 Tris, adjusted to pH 6.0 with 1 MES, and 600 mOsM, with D-sorbitol. The pipette solution contained 70 mM K gluconate, 10 mM KCl; 100 nM Ca^2+^ was adjusted with 1 mM BAPTA and 0.475 mM CaCl_2_, pH 7.2 with 2 mM Tris, 1 mM MES.

**Figure 3:**
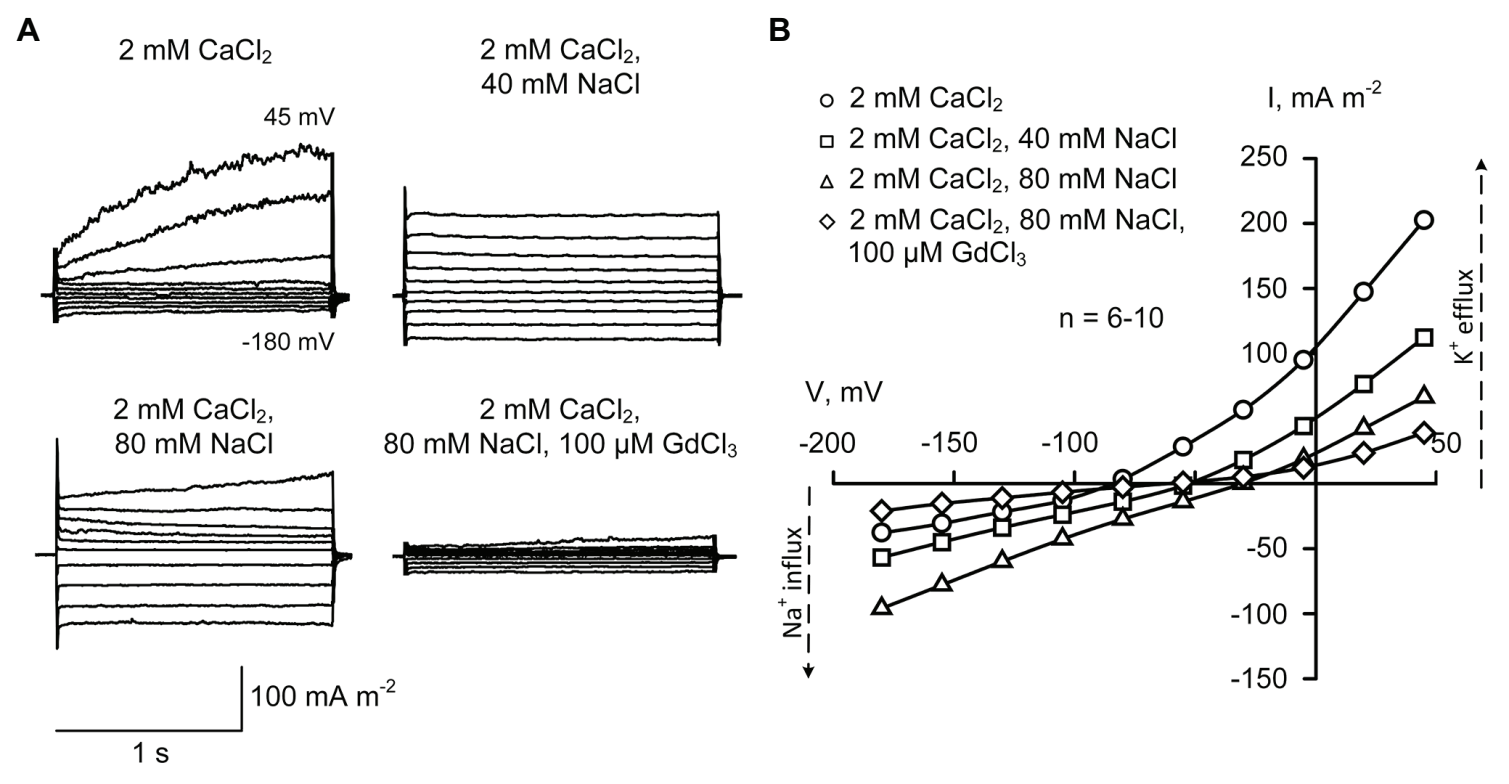
Typical plasma membrane currents **(A)** and mean current-voltage curves **(B)** in *H. annuus* L. root protoplasts recorded in the presence of 2 mM CaCl_2_ in the bathing solution at different levels of external NaCl (40 and 80 mM). Current-voltage curves were plotted by mean values (*n* = 6–10; errors are not shown in the figure). Statistically significant (*p* < 0.01; ANOVA test) difference between “2 mM CaCl_2_” (circles) and “40 mM NaCl” (squares) was found at all voltage values apart from −105 mV. The difference between “2 mM CaCl_2_” (circles) and “80 mM NaCl” (triangles) was statistically significant at all voltage values (*p* < 0.01; ANOVA test). The standard bathing solution contained (in mM): 0.3 KCl, 2 Tris, adjusted to pH 6.0 with 1 MES, and 600 mOsM, with D-sorbitol. The pipette solution contained 70 mM K gluconate, 10 mM KCl; 100 nM Ca^2+^ was adjusted with 1 mM BAPTA and 0.475 mM CaCl_2_, pH 7.2 with 2 mM Tris, 1 mM MES. 100 μM GdCl_3_ was added to the bathing solution on the background of 2 mM CaCl_2_ and 80 mM NaCl for 5 min before recording current-voltage curves.

**Figure 4:**
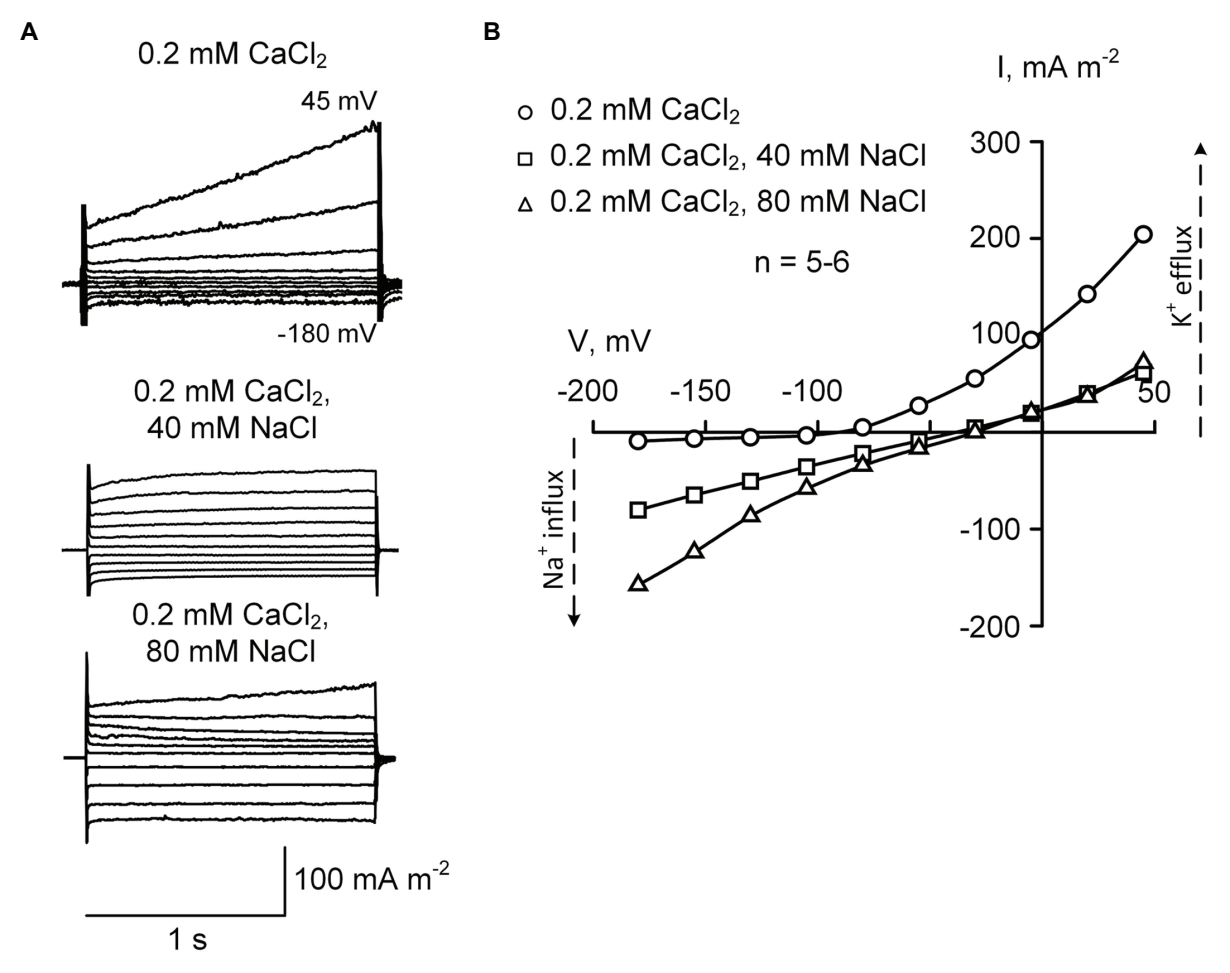
Typical plasma membrane currents **(A)** and mean current-voltage curves **(B)** in *H. annuus* L. root protoplasts recorded in the presence of 0.2 mM CaCl_2_ in the bathing solution at different levels of external NaCl (40 and 80 mM). Current-voltage curves were plotted by mean values (*n* = 5–6; errors are not shown in the figure). The difference between “0.2 mM CaCl_2_” (circles) and “40 mM NaCl” (squares) as well as between “0.2 mM CaCl_2_” (circles) and “80 mM NaCl” (triangles) was statistically significant at all voltage values (*p* < 0.01; ANOVA test). Statistically significant (*p* < 0.01; ANOVA test) difference between “40 mM NaCl” (squares) and “80 mM NaCl” (triangles) was found at a voltage of more negative than −80 mV. The standard bathing solution contained (in mM): 0.3 KCl, 2 Tris, adjusted to pH 6.0 with 1 MES, and 600 mOsM, with D-sorbitol. The pipette solution contained 70 mM K gluconate, 10 mM KCl; 100 nM Ca^2+^ was adjusted with 1 mM BAPTA and 0.475 mM CaCl_2_, pH 7.2 with 2 mM Tris, 1 mM MES.

**Figure 5:**
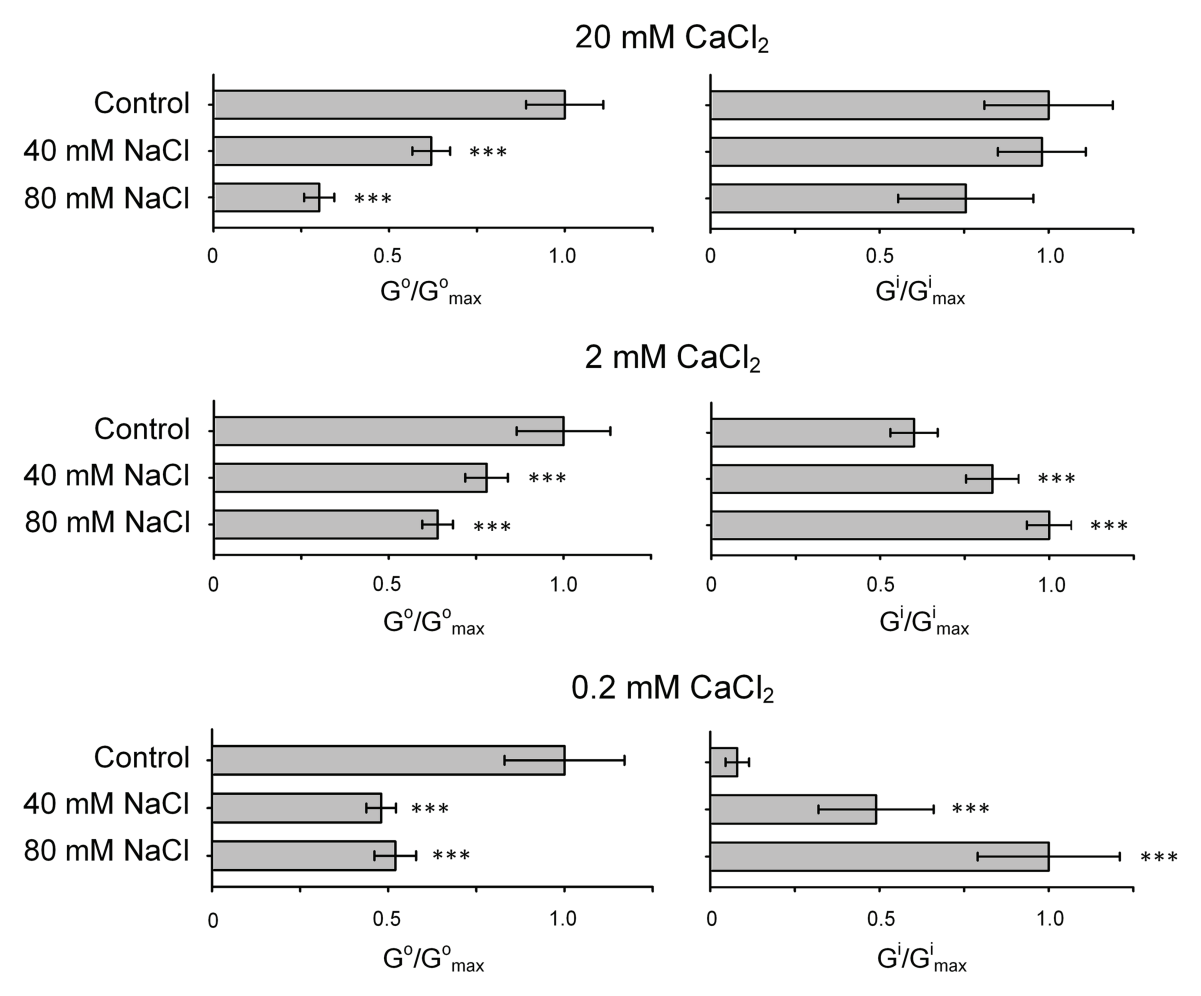
Changes in outwardly (G^o^) and inwardly (G^i^) directed conductance in *H. annuus* L. root protoplasts induced by addition of 40 or 80 mM NaCl to the external solution. The outwardly directed and inwardly directed conductance values were calculated using currents induced by voltage segment within the reversal potential to 50 mV and − 180 mV, respectively (based on IV curves shown in Figures 2–4). G_max_ is the maximal value of conductance measured calculated in an individual experiment (set of IV curves). Experimental conditions and ionic species in external and pipette solutions are same as in Figures 2–4. Data are mean ± SE (*n* = 5–11; ^***^p < 0.0001; ANOVA test; comparison to control; no significant difference where unmarked).

Protoplasts were patch-clamped in the sealing solution containing 20 mM CaCl_2_ and 0.3 mM KCl (pH 6.0) using pipettes filled with the solution comprising of 70 mM KGluconate and 10 mM KCl (pH 7.2, 100 nM Ca^2+^). High external Ca^2+^ allowed gigaseal formation (Demidchik and Tester, 2002), while high intracellular (pipette) K^+^ “mimicked” cellular K^+^ level (Demidchik, 2014). Potassium gluconate (70 mM) in the pipette solution was used instead of KCl to avoid Cl^−^ efflux currents, which can overlap with Na^+^ influx conductance. Gluconate is a poorly permeable organic anion that minimizes anion efflux currents in patch-clamped root protoplasts (Makavitskaya et al., 2018). In these conditions, a moderate negative inwardly directed current was measured (Figure 2A). This current was voltage-independent and sensitive to 100 μM Gd^3+^ (77.3 ± 4.5% decrease of the amplitude; ±SE; *n* = 5; data not shown). It showed very rapid (“instantaneous”) activation kinetics. When external CaCl_2_ was decreased from 20 to 0.2 mM, this current decreased by five times, demonstrating that it was mediated by Ca^2+^ influx (Figures 2–4, 5; *p* < 0.001; *n* = 5). These Ca^2+^ currents were similar to NSCC-mediated Ca^2+^ currents previously reported in *A. thaliana* root protoplasts (Demidchik et al., 2002). It should be noted that in Arabidopsis, NSCCs mediating these currents were Na^+^-permeable (Demidchik et al., 2002; Demidchik and Tester, 2002). Addition of NaCl to patch-clamped protoplasts in the presence of 20 mM extracellular Ca^2+^ did not induce inwardly directed current (as expected for Na^+^ influx NSCCs). The reversal potential was −91 ± 4 mV (20 mM CaCl_2_; ±SE; *n* = 11) and it was not modified by NaCl addition (Figure 2).

The outward current measured in the presence of 20 mM external CaCl_2_ was significantly blocked by the addition of NaCl to the bathing solution (Figure 2). The outwardly directed conductance dropped three times when 80 mM NaCl was added (Figure 5). In the conditions used in the work, the outward current could be mediated by K^+^ efflux through KORs or by Cl^−^ influx *via* anion channels (Demidcik et al., 2002, 2014; de Angeli et al., 2007; Demidchik, 2012; Hedrich, 2012). However, only K^+^ currents can be blocked by Na^+^ because the anion channels are insensitive to this and other alkali metals (Barbier-Brygoo et al., 2000). Moreover, the addition of K^+^ channel blocker TEA^+^ (30 mM TEACl) inside the patch-clamp pipette instead of 80 mM K^+^ (70 mM KGluc and 10 mM KCl) decreased the outward current by 8–9 times (*p* < 0.001; *n* = 5; data not shown) demonstrating that this current was mediated by KORs.

The time-dependent component of the outward K^+^ current was inhibited after the addition of NaCl to the bathing solution while instantaneous current remained very similar (Figure 2). It can be thus hypothesized that the residual outward current was mediated by anion channel-catalyzed Cl^−^ influx or K^+^ efflux *via* NSCCs (previously described in Shabala et al., 2006). The maximal reduction of the outward current was 4.3, as measured in the presence of 80 NaCl at 7.6 depolarizing pulses (Figure 2). This reduction was 3.2 times as calculated for 1.5-s-long segments of depolarising pulses (directly comparable with pulses used in Figures 3, 4). These results demonstrate a high sensitivity of KOR to Na^+^ and suggest a relatively low sensitivity of KOR to external Ca^2+^ in salt-tolerant sunflower.

### Sodium Influx Currents in Sunflower Root Protoplasts Under Low External Ca^2+^

Calcium ions are blockers of plant Na^+^-permeable NSCCs (Demidchik and Tester, 2002; Shabala et al., 2006). This may be the reason for no detection of Na^+^ influx conductance in the presence of 20 mM CaCl_2_ (Figure 2). However, the decrease of external Ca^2+^ from 20 to 2 mM (typical soil solution level of Ca^2+^; White and Broadley, 2003; Marschner, 2011) resulted in the increase in the inward Na^+^ current, which correlated with a shift of reversal potential to more positive values (from −86.6 ± 3.2 mV in control to −49.8 ± 2.5 mV at 40 mM NaCl and −30.1 ± 1.8 mV at 80 mM NaCl; ±SE; *n* = 6–11), consistent with currents being dominated by the movement of Na^+^ (Figure 3). This can be interpreted as weakening the Ca^2+^-induced blockade of the NSCCs. Sodium influx current showed an “instantaneous” kinetics and was voltage-independent.

The shift of the reversal potential in response to NaCl to more positive values decreased the KOR-mediated outwardly-directed currents (as the activation curve moved positive). Moreover, the decrease of the external CaCl_2_ destabilized patches and caused a breakdown at depolarization that did not allow depolarizing pulses longer than 1.5 s (note: 7.6 s-long pulses were applied at 20 mM CaCl_2_ to record full activation of KORs). In this regard, the measurements were limited to shorter segments of the outwardly-directed K^+^ currents (Figure 3; see also calculation of conductance change in Figure 5), and it was not possible to fully compare the data with those shown in Figure 2. The obtained data demonstrated that an addition of NaCl (both 40 and 80 mM), in the presence of 2 mM CaCl_2_, inhibited the outwardly-directed currents slightly weaker than in the presence of 20 mM CaCl_2_ (Figures 3, 5). The time-dependent component of the current was almost fully inhibited.

The addition of 100 μM Gd^3+^, which is a non-specific blocker of NSCCs and other plant cation channels (Demidchik and Maathuis, 2007) to the bathing solution containing 2 mM CaCl_2_ and 80 mM NaCl, caused a very strong inhibition of both inward and outward currents (5–6-fold decrease of currents; Figure 3). This indicates that both currents were mediated by cation channels (not by anion channels).

Lowering the external CaCl_2_ from 2 to 0.2 mM in the presence of 40 or 80 mM NaCl resulted in further increase in inwardly-directed voltage-independent Na^+^ current (Figure 4). The reversal potential values measured after the addition of 40 and 80 mM NaCl were − 37.4 ± 2.9 mV and −26.5 ± 3.2 mV, respectively (±SE; *n* = 5). These values were more positive compared to those measured at 2 mM Ca^2+^, suggesting that it was due to increased permeability to Na^+^ (in conditions of external 40 or 80 mM Na^+^, Na^+^ reversal potential is positive). The outwardly-directed K^+^ efflux conductance was equally blocked by 40 and 80 mM NaCl in the presence of 0.2 mM CaCl_2_, suggesting the saturation of the blockade at 40 mM NaCl or lower level of salt (Figures 4, 5). Interestingly, the time-dependent current component was almost fully blocked, when 40 or 80 mM NaCl were added on the background of 0.2 mM CaCl_2_.

## Discussion

Overall, data reported here demonstrate for the first time that *H. annuus* root plasma membrane has a set of ionic conductances dominated by NSCCs and KORs. Similar conductances were previously recorded in the plasma membranes of root protoplasts isolated from *A. thaliana* (Maathuis and Sanders, 2001; Demidchik et al., 2002, 2010; Demidchik and Tester, 2002; Shabala et al., 2006), *Thellungiella halophila* (Volkov et al., 2004; Volkov and Amtmann, 2006), *Pisum sativum* (Zepeda-Jazo et al., 2011), *T. aestivum* (Straltsova et al., 2015) and other species (Demidchik, 2014). To our knowledge, this work is the first patch-clamp and voltage-clamp study on sunflower. It should be noted that previous works have touched on the topic of sunflower electrophysiology only in terms of measurements of membrane potential (Stankovic et al., 1997).

In this investigation, the *Helianthus* Na^+^ influx currents were also measured and analyzed (Figures 2–4). These currents showed voltage-independent activation, lack of time-dependent component and high sensitivity to Gd^3+^. These properties are fully in line with the characteristics of Na^+^-permeable NSCCs previously measured in *A. thaliana* (Maathuis and Sanders, 2001; Demidchik and Tester, 2002; Shabala et al., 2006) and *T. halophile* (Volkov et al., 2004; Volkov and Amtmann, 2006). However, sunflower Na^+^-permeable NSCCs showed a much weaker response to the decrease of extracellular Ca^2+^ as compared to *Arabidopsis* or *Thellungiella* in the range of physiological Ca^2+^ levels (2–0.2 mM). Thus, sunflower NSCCs has smaller Na^+^ current density (and potentially lower number of channels per same membrane area) than *Arabidopsis* or *Thellungiella* at physiological extracellular [Ca^2+^], potentially preventing toxic Na^+^ influx and cell reactions induced by NaCl. This makes it possible to assume that Ca^2+^ could cause greater inhibition of NSCCs in sunflower roots. Interestingly, the response of Na^+^ influx to Ca^2+^ correlated well with Ca^2+^-induced protection of root growth in sunflower seedlings treated with NaCl at different external [Ca^2+^] (Figure 1). Growth inhibition by 80 mM NaCl was prevented by 2 mM CaCl_2_ while the treatment with 0.2 CaCl_2_ was not effective (Figure 1).

Results presented here also demonstrate a high sensitivity of KOR to Na^+^ and suggest a relatively low sensitivity of KOR to external Ca^2+^ in salt-tolerant sunflower. Similar sensitivity to external Na^+^ is known for animal KORs, such as Kv2.1 and related to Na^+^ reaction with the high and low affinity Na^+^ binding sites in Kv2.1 channel (Kiss et al., 1998). Potassium outwardly-directed conductances mediated by KORs in salt-tolerant *T. halophila* decreased 1.5–1.7 times after the addition of 100 mM external Na^+^ (Volkov et al., 2004; Volkov and Amtmann, 2006). In salt-sensitive species *A. thaliana*, this blockade was 1.3–1.9 times both in root epidermis and leaf mesophyll cells (showing a tendency to increase with an increase in the concentration of extracellular Ca^2+^; Shabala et al., 2006). From the present findings, we hypothesize that enhanced sensitivity of K^+^ efflux system to Na^+^ can play an important role for adaptation because this will decrease K^+^ loss under salinity conditions. It fits well within the hypothesis that maintaining a high K^+^/Na^+^ ratio in plant cells and prevention of K^+^ efflux under salt stress are key mechanisms of salt tolerance in higher plants (Shabala and Cuin, 2012; Demidchik et al., 2014, 2018).

Intriguingly, K^+^ outwardly directed conductance in sunflower showed greater Na^+^ sensitivity at higher extracellular CaCl_2_ levels that can have a positive effect in conditions of salinity (as cells will lose less K^+^; Figure 5). This can be explained by the influence of CaCl_2_ on the Na^+^-induced blockade of KORs in the case of measurements which were carried out at 20 and 2 mM external Ca^2+^. In animal plasma membrane K^+^ channels, Na^+^ can compete with K^+^ for binding sites within a pore region modulating channel characteristics and functions in Ca^2+^-dependent manner (Kiss et al., 1998; Sauer et al., 2013). In animals, Ca^2+^ modifies the K^+^ channel activity *via* action on the surface charge, reaction with the specific binding sites at extracellular loops, effect on the EF-hands and calmodulin binding sites at cytosolic side (Shah et al., 2006). We hypothesize that the elevated extracellular Ca^2+^ controls the Na^+^ block of the sunflower K^+^ channel by increasing Na^+^ sensitivity. Interestingly, Lemtiri-Chlieh et al. (2020) have recently reported that divalent cation Mg^2+^ added to the pipette solution can change both the activity of leaf NSCCs and their sensitivity to Gd^3+^, suggesting sophisticated interactions of cations within the NSCC complex.

Involvement of root KORs (potentially encoded by Shaker-type GORK) to NaCl responses and salt stress adaptation have been demonstrated in a number of species (Adem et al., 2020). It is a redox-dependent phenomenon as GORK is additionally activated by ROS (Demidchik et al., 2010). Potassium loss *via* GORK triggered by depolarization and ROS can lead to ionic disequilibrium, induction of autophagy, and programmed cell death (Demidchik et al., 2010, 2018). Enhanced blockade of KOR by Na^+^ will be the simplest and “economical” mechanism for preventing K^+^ loss that will retain the greatest amount of metabolic energy for adaptation in salinity conditions. The cell’s energy balance has recently been recognized as one of the main salt stress targets (Tyerman et al., 2019). Thus targeting KORs and their Na^+^ sensitivity regions to save energy for reparation needs offers high hopes for generation of salt-tolerant varieties by molecular breeding techniques.

In conclusion, the data presented here strongly suggest that the moderate resistance of sunflower to NaCl stress is programmed at potassium and non-selective channel level *via* the sensitivity of ion channels to Ca^2+^ and Na^+^.

## Data Availability Statement

The raw data supporting the conclusions of this article will be made available by the authors, without undue reservation.

## Author Contributions

VD was responsible for research supervision, experimental design, management of experiments, data analysis, and writing the manuscript. SB and MY were involved in the preparation of plant material, research supervision, and design of experiments. PH, IN, YT, XH, and MK carried out electrophysiological experiments. VS and AV conducted hydroponics studies. AS and IS carried out routine cultivation of sunflower seedlings, maintained patch-clamp equipment, and participated in manuscript preparation. All authors contributed to the article and approved the submitted version.

### Conflict of Interest

The authors declare that the research was conducted in the absence of any commercial or financial relationships that could be construed as a potential conflict of interest.
